# Synthesis, Characterization, and Evaluation of Folic Acid Release Ability of Acrylamide–Acrylic Acid Hydrogels and Acrylamide–Acrylic Acid/Functionalized Carbon Nanotube Nanocomposite Hydrogels

**DOI:** 10.3390/ijms26209847

**Published:** 2025-10-10

**Authors:** Karina Sandoval-García, Jorge A. Cortés-Ortega, Edgar B. Figueroa-Ochoa, Víctor H. Antolín-Cerón, Sergio M. Nuño-Donlucas

**Affiliations:** 1 Departamento de Física, Centro Universitario de Ciencias Exactas e Ingenierías, Universidad de Guadalajara, Guadalajara 44430, Mexico; karina.sandoval@academicos.udg.mx; 2 Departamento de Química, Centro Universitario de Ciencias Exactas e Ingenierías, Universidad de Guadalajara, Guadalajara 44430, Mexico; jorcortes@hotmail.com (J.A.C.-O.); benjamin.figueroa@academicos.udg.mx (E.B.F.-O.); 3 Departamento de Ciencias Básicas Aplicadas e Ingeniería, Centro Universitario de Tonalá, Universidad de Guadalajara, Tonala 45425, Mexico; victor.antolin@academicos.udg.mx; 4 Departamento de Ingeniería Química, Centro Universitario de Ciencias Exactas e Ingenierías, Universidad de Guadalajara, Guadalajara 44430, Mexico

**Keywords:** hydrogels, carbon nanotubes, nanocomposites, acrylamide, acrylic acid, folic acid

## Abstract

Hydrogels of acrylamide (AM)–acrylic acid (AA) and nanocomposite hydrogels of AM–AA and carbon nanotubes (CNTs) functionalized with acyl chloride groups (CNTs_OxCl_) were synthesized and characterized, and their ability to release folic acid was analyzed. Both hydrogel types were synthesized via redox polymerization. CNTs were prepared via chemical vapor deposition. The prepared samples were analyzed via transmission electron microscopy, Raman spectroscopy, X-ray photoelectron spectroscopy, differential scanning calorimetry, and field-emission scanning electron microscopy. Their swelling ability and their mechanical properties (compression tests) were determined at room temperature ~298.15 K, whereas their ability to release folic acid was studied using UV–VIS spectroscopy. The equilibrium swelling of the AM–AA hydrogels was greater than that of the AM–AA/CNTs_OxCl_ nanocomposite hydrogels prepared at the same monomeric relation (wt%), whereas the Young moduli of these nanocomposite hydrogels were higher than that of AM–AA hydrogels. For the AM–AA/CNTs_OxCl_ nanocomposite hydrogels, polymer chains containing AM and AA units were grafted to CNTs_OxCl_. The glass–transition temperatures of AM–AA nanocomposite hydrogels were higher than that of AM–AA hydrogels. Folic acid release from the AM–AA hydrogels and AM–AA/CNTs_OxCl_ nanocomposite hydrogels was successfully adjusted using the Weibull model.

## 1. Introduction

The design of stimuli-responsive drug delivery systems (DDS) has attracted considerable interest in recent years. DDS are sensitive to external stimuli and can provide targeted and controlled drug delivery. Among these, hydrogels have been highlighted for their biocompatibility, nontoxicity, high water content, swelling capacity, and compatibility with several cellular lines [1,2].

Depending on their nature or the type of energy applied, the stimuli that trigger drug release from a specific DDS can be classified into three types: (i) chemical (pH and ionic strength), (ii) physical (temperature, ultrasound, osmotic pressure, and magnetic field), and (iii) biological (endogenous receptors and enzymes) stimuli [3].

Among thermoresponsive polymers, hydrogels are of particular interest owing to their permeability, allowing the diffusion of water or biological fluids, which can be exploited in DDS [4]. Thermoresponsive polymers can be classified into two groups: those with a lower critical solution temperature (LCST), and those with an upper critical solution temperature (UCST) [5]. Thermoresponsiveness involves a sharp transition within a narrow temperature range [6]. Hydrogels with UCST shrink below this temperature but swell upon heating. Their use as drug carriers is rarely reported because such materials are uncommon, particularly under physiological conditions [7]. Nevertheless, UCST hydrogels are of interest for treating fever-related diseases, where elevated body temperatures can induce drug release [8].

The poly(acrylic acid–co–acrylamide) [P(AA–co–AM)] copolymer hydrogel is both pH- and temperature-responsive and exhibits a UCST that decreases with increasing pH [9]. Several studies have characterized P(AA–co–AM) hydrogels and evaluated their drug delivery potential. Ding et al. prepared P(AA–co–AM) hydrogels that were combined with biocompatible SiO_2_ and polypyrrole (PPy) to obtain a light-, thermal-, and pH-responsive DDS and evaluated their ability to release 5–fluorouracil (5–FU), an anticancer drug. In this system, PPy can convert near-infrared (NIR) irradiation into heat, maintaining the temperature in the UCST range of P(AA–co–AM), while the SiO_2_ particles reduce burst release, achieving 83.9% cumulative release of 5–FU at 6 h at pH 7.4 [10]. Ray et al. [11] synthesized both P(AA–co–AM) normal hydrogels and P(AA–co–AM) hydrogel nanoparticles via free-radical polymerization using N,N–methylene–bis–acrylamide (MBA) as the cross-linker and potassium persulfate as the initiator. Their results showed that the nanoparticles were more effective drug carriers for colon-targeted 5-FU release than the P(AA–co–AM) normal hydrogel. Similarly, Thippeswamy et al. synthesized P(AA–co–AM) hydrogels using MBA as the crosslinking agent, and potassium persulfate and sodium metabisulfite were used as the initiators. They evaluated the release of moxifloxacin hydrochloride as a function of pH and observed a higher release at pH 7.4 (99%) than that at 1.2 (18%), with the swelling ratio also increasing with temperature and the Higuchi model successfully describing the release kinetics [12].

Although P(AA–co–AM) hydrogels have been implemented as drug carriers, several challenges remain. The mechanical properties of hydrogels play a crucial role in drug delivery applications. In P(AA–co–AM) hydrogels synthesized via radical polymerization, increasing the cross-linker agent concentration improves the mechanical properties; however, it has a negative impact on the swelling capacity due to decreased porosity, which may in turn slow drug release by limiting diffusion through capillary channels [13].

Carbon nanotubes (CNTs) possess outstanding chemical, mechanical, electrical, and thermal properties, and are considered as the most robust materials known in nature [14]. Their high axial strength [15] and high Young’s modulus, in the range of 0.32–1.47 TPa for single-walled CNTs (SWCNTs) and 0.27–0.95 TPa for multi–walled CNTs (MWCNTs) [16] make them excellent reinforcing agents. CNTs can be dispersed within polymers (used as a matrix) to prepare polymer nanocomposites with enhanced performance. CNT–polymer nanocomposites and smart hydrogels exhibit significant potential for drug delivery and constitute a promising strategy for treating lethal diseases [17].

The use of CNTs as a reinforcing agent to enhance the mechanical properties of poly(acrylamide)-based hydrogels has been well documented. Feng et al. prepared poly(acrylamide)–MWCNT nanocomposite hydrogels via radiation-induced polymerization and evaluated their mechanical properties. Compressive tests revealed extremely high mechanical strengths for these composites compared with the pristine poly(acrylamide) hydrogel, even when the content of MWCNTs was as low as 0.05 wt%, while maintaining a high water content (>80 wt%). These nanocomposites also exhibited higher elastic moduli compared with pure poly(acrylamide) hydrogels [18]. Takada et al. [19] evaluated the tensile elastic modulus and swelling of polyacrylamide (PAAm)/polyacrylic acid (PAAc) nanocomposite hydrogels (with two structures: a random copolymer and an interpenetrating network (IPN)) reinforced with MWCNTs. Both structures showed higher tensile elastic moduli than those of the pristine PAAm/PAAc hydrogels and decreased swelling. Furthermore, the structure of the nanocomposite hydrogels influences their mechanical response, exhibiting a higher tensile modulus than random copolymers. Kanca et al. [20] prepared nanocomposite hydrogels by incorporating MWCNTs into a covalently bonded PAAm/PAAc hydrogel, with scanning electron microscopy revealing a homogenous MWCNTs distribution. Moreover, compression tests at 10–40% strain (strain rate 1/min) in phosphate-buffered saline at room temperature revealed that the nanocomposite hydrogels (prepared with 0.5% and 1.0% MWCNTs) had higher compressive moduli than that of pristine PAAm/PAAc hydrogels, confirming their potential as cartilage substitutes.

The choice of drug is also critical in drug release studies. Folic acid (FA) is a particularly attractive biological molecule, because it targets folic acid receptors, which are often overexpressed on the surface of cancer cells [21]. The use of FA as a “Trojan horse” strategy for drug delivery to cancer cells has been explored previously [22], motivating the design of DDSs loaded with FA.

In this study, AM–AA hydrogels and AM–AA/CNTs_OxCl_ nanocomposite hydrogels were synthesized and characterized, and their capacity to release FA was evaluated. CNTs were synthesized via chemical vapor deposition (CVD), purified with overheated steam, partially oxidized through acid treatment, and chemically functionalized with oxalyl chloride (OxCl) to insert acyl chloride functionalities on their walls. Functionalized CNTs (CNTs_OxCl_) were used to prepare nanocomposite hydrogels, and the AM–AA and AM–AA/CNTs_OxCl_ nanocomposite hydrogels were prepared via redox polymerization. The morphologies of the purified CNTs, partially oxidized CNTs, and functionalized CNTs were examined using transmission electron microscopy (TEM). Raman spectroscopy was used to study the vibrational behaviors of the partially oxidized and functionalized CNTs, as well as the AM–AA hydrogels and AM–AA/CNTs_OxCl_ nanocomposite hydrogels. Swelling data were fitted using a second-order kinetic model. All hydrogels were further analyzed by X-ray photoelectron spectroscopy (XPS), differential scanning calorimetry (DSC), and field-emission scanning electron microscopy (FE–SEM), and their mechanical properties were evaluated using compression tests. FA release from the prepared hydrogels was examined using various kinetic models.

## 2. Results and Discussion

**Figure 1:**
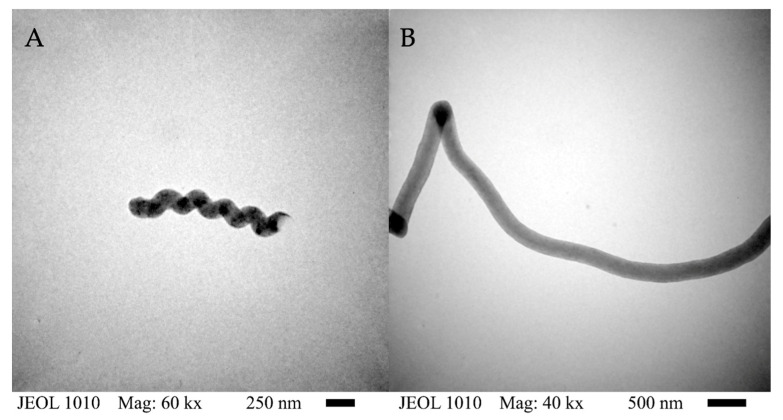
TEM micrographs of a helical carbon nanotube (HCNT) (**A**) and a tubular CNT (**B**). Both nanostructures were detected in a CNTs_pur_ sample.

Figure 1 shows the TEM micrographs of the CNTs_pur_ sample. In Figure 1A, a helical CNT is observed, whereas in Figure 1B, a straight CNT is detected. Both nanostructure types were observed in the same CNTs_pur_ sample. Structural analysis of the CNTs employed in the preparation of the nanocomposite hydrogels is important because, owing to the high anisotropy of graphite, the mechanical properties of CNTs strongly depend on their morphology [23]. The helical CNTs reach lengths greater than 1 μm, with diameters of approximately 100 nm. Such dimensions strongly suggest the presence of helical MWCNTs (HMWCNTs), consistent with our previously reported findings using a similar synthetic procedure [24]. Furthermore, the lengths of the straight CNTs are larger than several microns, with diameters of approximately 80 nm. The coexistence of helical and straight CNTs synthesized via CVD using Fe as a catalyst has been reported previously [25]. Theoretical studies and systematic measurements have determined that the Young’s modulus of HMWCNTs typically ranges from 300 to 1000 GPa, though values up to 1.28 TPa have been also reported [26]. For straight MWCNTs, the Young’s modulus is in the range from 1.05 to 1.58 TPa [27]. Owing to their curved sections, helical CNTs are expected to be more reactive than straight CNTs, because of the sensitivity detected at their curved tips.

To introduce hydroxyl and carboxyl groups into the CNTs_pur_ walls, partial oxidation was performed as described in the materials and methods section. Following the methodologies of Xia et al. [28] and Silva-Jara et al. [29], the amounts of carboxyl and hydroxyl groups were calculated, resulting 40.5 and 13.3 wt% per gram of CNTs_poxi_, respectively.

**Figure 2:**
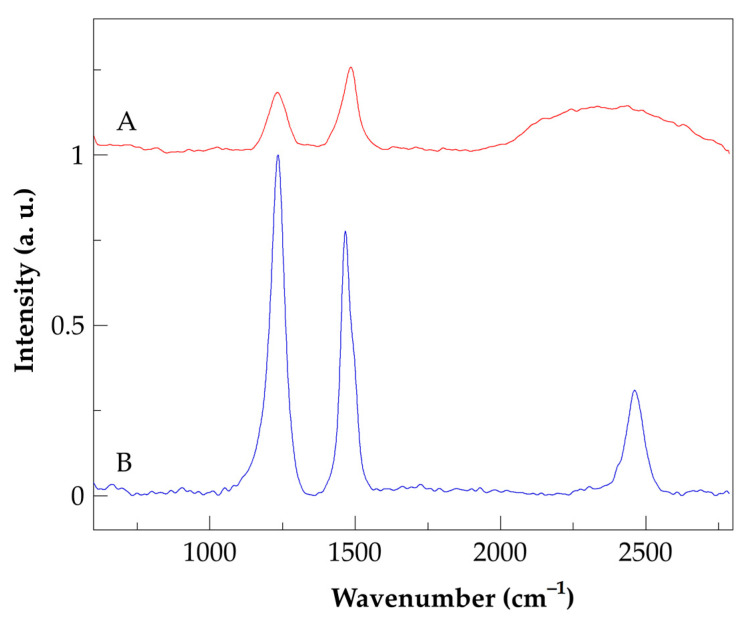
Raman spectra of CNTs_poxi_ (A) and CNTs_OxCl_ (B).

Figure 2 shows the Raman spectra of CNTs_poxi_ (Figure 2A) and CNTs_OxCl_ (Figure 2B), revealing three bands in both spectra. Table 1 lists the wavenumbers of the D, G, and G’ bands, the area of the G-band, and the I_D_/I_G_ ratio determined from the scattering intensities of the D- and G-bands. At low frequencies, a disorder-induced band (D-band) is detected (at 1233 cm^−1^ in CNTs_poxi_ and 1235 cm^−1^ in CNTs_OxCl_). A D-band is produced when a double-resonance process occurs, owing to elastic and inelastic scattering caused by defects and phonons, respectively [30]. In the Raman spectra of SWCNTs bundles, the D-band is observed between 1250 and 1450 cm^−1^ and depends on the mean nanotube diameter [31]. In MWCNTs, the D-band shifts to lower frequencies [32]. This finding confirms the presence of MWCNTs in the CNTs_poxi_ and CNTs_OxCl_ samples, consistent with the micrographs of CNTs_poxi_ shown in Figure 1. The bands at 1485 cm^−1^ (CNTs_poxi_) and 1466 cm^−1^ (CNTs_OxCl_) are attributed to the graphite band (G-band), caused by a first-order Raman effect. The G-band of different carbonaceous materials is typically detected in the region of 1450–1750 cm^−1^ [33]. In CNTs, the G-band involves an optical phonon mode between two carbon atoms in one graphene unit cell, which constitutes the basic structure of a graphene sheet. This band is caused by high ordered carbon structures like those that form the walls of the CNTs. The shift in the G-band to lower frequencies in CNTs_OxCl_ suggests a modification of the vibrational response of CNTs_OxCl_, caused by the presence of guest molecules with different electronic characteristics. According to the chemical functionalization route used in this study, this change can be attributed to the presence of acyl chloride groups on the CNTs_OxCl_ walls. At higher frequencies, a G’-band is detected at 2436 cm^−1^ for CNTs_poxi_ and 2461 cm^−1^ for CNTs_OxCl_. For carbonaceous materials, the G’-band is observed as a broad peak in the 2400–2800 cm^−1^ region [33], arising from a double-resonance process. G’-band is an overtone of the D-band and is observed in the spectrum of materials with sp^2^ bonded carbon atoms. Similarly to the D-band, the G’-band is sensitive to the chirality and diameter of CNTs. The clear differences in intensity and broadness of the G’-band detected in the spectra of CNTs_poxi_ and CNTs_OxCl_ are caused by the different structural arrangements of both CNT types. The I_D_/I_G_ ratio is higher for CNTs_OxCl_ than for CNTs_poxi_, indicating a decrease in the order and crystallinity after functionalization. Fantini et al. attributed such increases in disorder to the formation of structural defects caused by the attachment of chemical groups to the CNTs walls [34]. This supports the conclusion that the presence of acyl chloride groups in the CNTs_OxCl_ walls increases the disorder of the walls. Our previous study involving Fourier transform infrared spectroscopy (FT–IR) confirmed the insertion of acyl chloride groups onto partially oxidized CNTs following a similar chemical route to that used in this work [35].

**Figure 3:**
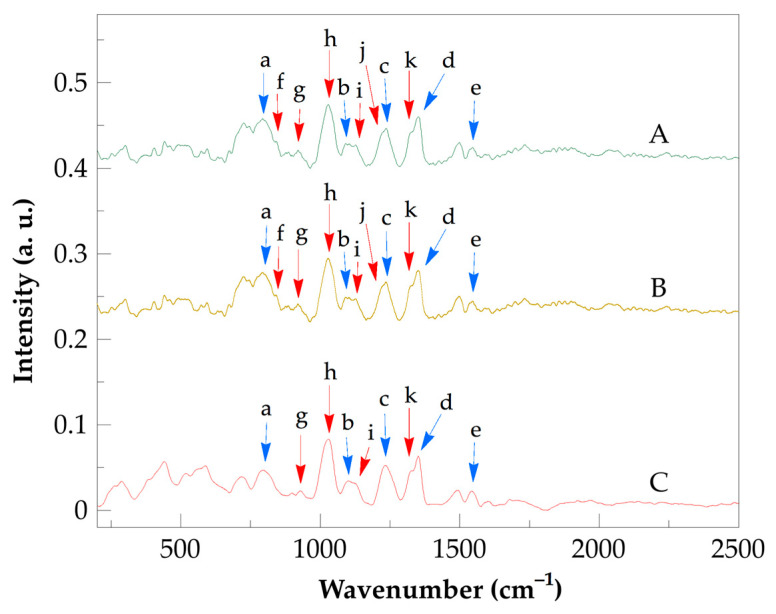
Raman spectra of Hydro 1 (A), Hydro 2 (B), and Hydro 3 (C).

Figure 3 shows the Raman spectra of dry samples of hydrogels Hydro 1 (Figure 3A), Hydro 2 (Figure 3B), and Hydro 3 (Figure 3C) in the region of 200–2500 cm^−1^. The vibrational band assignments for the polyacrylamide chains (blue arrows) were based on a previous report [36]. In the spectrum of Hydro 3, characteristic peaks are observed at 796 cm^−1^ (CH_2_ rocking vibration), 1101 cm^−1^ (C–C skeletal stretching), 1233 cm^−1^ (NH_2_ wagging), 1352 cm^−1^ (CH_2_ wagging), and 1544 cm^−1^ (amide I, C=O stretching) [37]. Similar spectral contributions are detected in Hydro 2 and Hydro 1 at nearly the same frequencies. For poly(acrylic acid) chains (red arrows), most contributions appear as shoulders adjacent to the polyacrylamide chain bands, except for two peaks. In the spectrum of Hydro 1, a weak band at 921 cm^−1^ is assigned to the stretching vibrations of the carboxylic acid dimer, and an intense band at 1029 cm^−1^ is attributed to CH_2_ rocking [38]. Other peaks assigned to the vibrations of the poly(acrylic acid) chains are detected at 843, 1127, 1224, and 1326 cm^−1^, attributed to C–COOH stretching [38], C-CH_2_ stretching, C–O stretching coupled with O–H in-plane bending, and –CCO stretching [39], respectively. Hydro 2 shows a very similar spectral pattern. Conversely, Hydro 3 exhibits a shift in the carboxylic acid dimer band to 930 cm^−1^ and the disappearance of the weak shoulders attributed to C–COOH stretching and C–O/O–H coupled vibrations in plane bending. These changes may be due to the higher poly(acrylic acid) chain content of Hydro 3, which modifies the vibrational frequencies of some sensitive groups. Additionally, other peaks (not marked with arrows) at wavenumbers between 250 and 750 cm^−1^ were detected (more clearly in the spectrum 3C). Thus, from low to high wavenumbers, peaks detected at 288, 441, 558, and 718 cm^−1^ were assigned to C–C bending of aliphatic chain, C–C=O bending, –COOH skeletal vibration, and O–C–O bending of carboxilate functionality, respectively. The coexistence of Raman bands due to the vibrations of the polyacrylamide and poly(acrylic acid) chains confirms the successful synthesis of the AM–AA hydrogels. Table 2 lists the most significant bands described and marked with a blue or red arrow.

**Figure 4:**
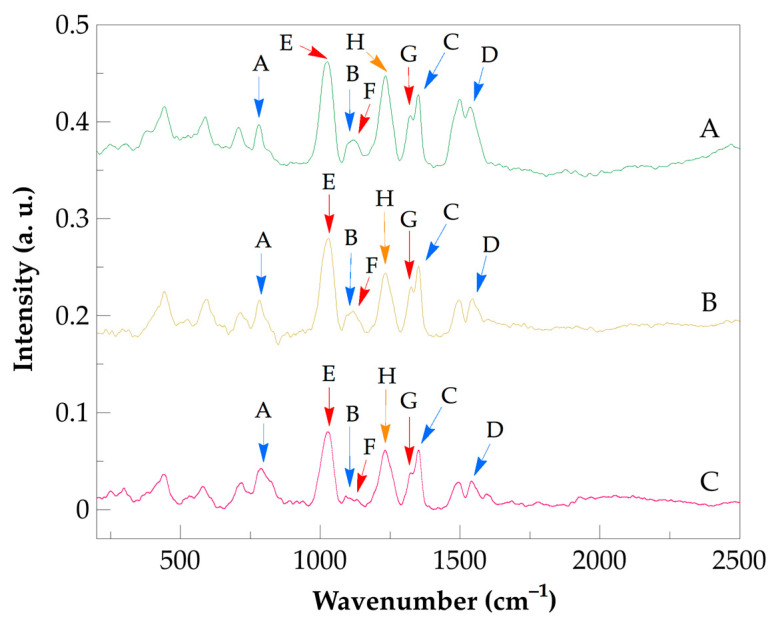
Raman spectra of Nano Hydro 1 (A), Nano Hydro 2 (B), and Nano Hydro 3 (C).

Figure 4 presents the Raman spectra of dry samples of the nanocomposite hydrogels Nano Hydro 1 (Figure 4A), Nano Hydro 2 (Figure 4B), and Nano Hydro 3 (Figure 4C) recorded in the 200–2500 cm^−1^ region. Comparing the spectrum of Nano Hydro 1 with Hydro 1 (shown in Figure 3A), several differences are observed; although the majority of spectral contributions are similar, significant differences are also evident: (i) the amide I band (C=O stretching vibration of the amide functionality of the polyacrylamide chains) is detected at 1536 cm^−1^ in Nano Hydro 1, lower (red shift) than 1546 cm^−1^ in Hydro 1. This red shift indicates hydrogen bonding between the amide groups of the polyacrylamide chains and the hydroxyl or carboxyl groups (which did not react with OxCl) in the walls of CNTs_OxCl_, consistent with previously reported Raman spectroscopy findings [40]. (ii) The carboxylic acid dimer band observed at 921 cm^−1^ in the spectrum of Hydro 1 disappear in Nano Hydro 1, indicating no self-association of the carboxylic acid groups of the poly(acrylic acid) chains is developed. Instead, new hydrogen bonds likely form between the carboxyl groups of the poly(acrylic acid) chains and hydroxyl and carboxyl groups of CNTs_OxCl_ in Nano Hydro 1. Supporting this, the –CCO stretching band shifts from 1326 cm^−1^ (Hydro 1) to 1322 cm^−1^ (Nano Hydro 1). It is also possible that in Nano Hydro 1, a high number of carboxylate ions included in the AA units are not associated via hydrogen bonds, because the shift in –CCO stretching band is small. It is important to mention that the formation of the dimer detected in Hydro 1 prevents the formation of carboxylate ions in the hydrogels prepared without CNTs_OxCl_. (iii) The most significant change is the appearance of the 1234 cm^−1^ band (orange arrow). In the spectrum of Hydro 1, a double band at 1236 cm^−1^ with a shoulder at 1224 cm^−1^ is detected, with a lower intensity than that of the nearby band at 1352 cm^−1^. In the spectrum of Nano Hydro 1, only one peak is observed at 1234 cm^−1^, with a higher intensity than that of the nearby band at 1353 cm^−1^, suggesting additional vibrations increasing the intensity of the 1234 cm^−1^ band. As shown in Figure 2, a D-band is detected at 1235 cm^−1^ in the spectrum of CNTs_OxCl_. Therefore, we consider that the increased intensity of the 1234 cm^−1^ band in the spectrum of Nano Hydro 1 is because of additional vibrations originating due to the presence of CNTs_OxCl_. Similar changes are observed in the spectra of Nano Hydro 2 and Nano Hydro 3. Table 3 shows the bands described and marked with a blue, red, or orange arrow. It is important to mention that Raman spectroscopy has well-known limitations (such as low sensitivity caused by weak spontaneous Raman signal or the presence of fluorescence that masks some signals), which may limit the analysis presented.

The total conversion (%) at the end of the polymerization for AM–AA hydrogels and AM–AA/CNTs_OxCl_ nanocomposite hydrogels was calculated by gravimetry, resulting in 90%, 97% and 95% for Hydro 1, Hydro 2, and Hydro 3, respectively. In addition, for Nano Hydro 1, Nano Hydro 2, and Nano Hydro 3, the results were 91%, 97% and 94%, respectively. These results indicate that the chemical route used to obtain the synthesized hydrogels was successful.

**Figure 5:**
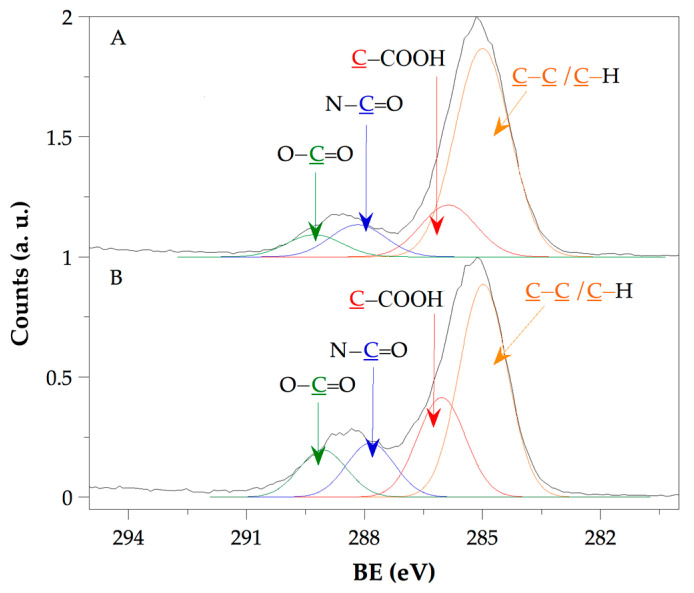
C*1s* core-level normalized spectra of Nano Hydro 2 (A) and Hydro 2 (B).

**Scheme 1:**
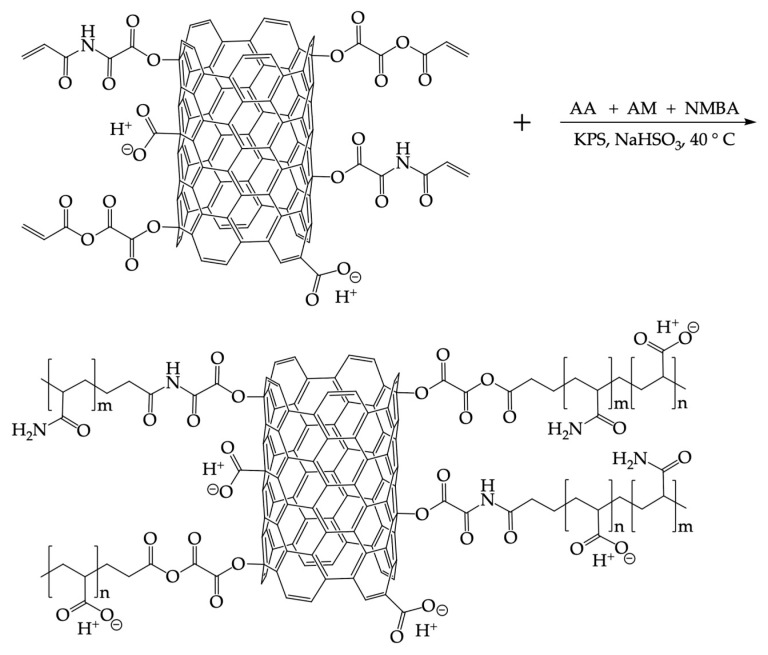
Polymerization reaction of AA and AM monomers on CNTs_OxCl_ previously decorated with AA and AM units.

Figure 5 shows the *C1s* core level normalized spectra of Nano Hydro 2 (Figure 5A) and Hydro 2 (Figure 5B), peak-fitted using the active background method included in AAnalyzer v 1.42 software [41]. The components and their atomic concentrations of Hydro 2 involve C–C/C–H bonds at 285.0 eV [42] (39.5%), CH–COOH bonds at 285.8 eV [43] (19.3%), –N–C=O bonds at 287.9 eV [44,45] (9.6%), and –O–C=O bonds at 289.0 eV [42] (7.7%). Furthermore, the components of Nano Hydro 2 and their atomic concentrations include C–C/C–H bonds at 285.0 eV [42] (39.4%), CH–COOH bonds at 285.9 eV [43] (9.2%), –N–C=O bonds at 288.1 eV [45] (6.1%), and –O–C=O bonds at 289.3 eV [42] (3.4%). The reduced molar concentrations and slight shifts to higher binding energies of the CH–COOH, –O–C=O, and –N–C=O bonds in Nano Hydro 2 compared with Hydro 2 suggest that chemical reactions occur, in which these chemical functionalities participate. Similar spectral behaviors have been reported previously [46,47]. Acyl chloride groups react with nucleophiles bonded to hydrogen atoms during addition-elimination reactions. Thus, based on the obtained results, we consider that the acyl chloride groups inserted on the CNTs_OxCl_ walls react with the nitrogen atom of the amine bonds that form part of the primary amide functionality inserted on the acrylamide monomer, producing secondary amide groups and grafting these monomeric molecules onto CNTs_OxCl_. Because the acyl chloride functionality also reacts with water (the solvent used to prepare the hydrogels and nanocomposite hydrogels) to produce carboxyl groups, the reaction should be carried out at the beginning of the nanocomposite hydrogel synthesis. In parallel, the hydroxyl groups inserted on the CNTs_OxCl_ walls can react with the carboxyl groups that form part of either the acrylic acid monomer or poly(acrylic acid) chains to produce ester groups. Through this chemical path, monomeric acrylic acid units or poly(acrylic acid) oligomers (more available at the beginning of the nanocomposite hydrogel synthesis) are grafted onto the CNTs_OxCl_ walls. Scheme 1 presents the chemical reaction described showing the polymeric chains formed by AM and AA units grafted onto functionalized CNTs_OxCl_. The left-hand side of this chemical reaction shows CNTs_OxCl_ decorated with AA and AM monomeric units. The right-hand side shows the final structure, which was embedded within the gel network. Notably, owing to the synthesis of AM–AA hydrogels and AM–AA/CNTs_OxCl_ nanocomposite hydrogels via radical polymerization, the grafted chains on CNTs_OxCl_ contain randomly ordered AA and AM units.

**Figure 6:**
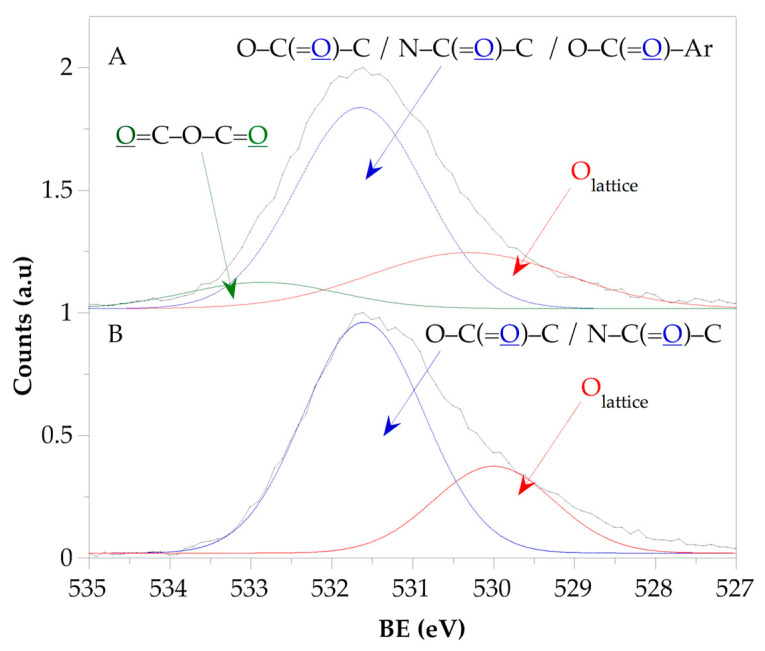
O*1s* core-level normalized spectra of Nano Hydro 2 (A) and Hydro 2 (B).

Figure 6 shows the O*1s* core-level normalized spectra of Nano Hydro 2 (Figure 6A) and Hydro 2 (Figure 6B), with the components of the *O1s* spectrum of Hydro 2 being observed at two positions. The components and their atomic concentrations are C_lattice_ at 530.6 eV [48] (3.5%) and O–C(=O)–C [49]/N–C(=O)–C [43] at 531.5 eV (4.5%). Conversely, the components of the *O1s* spectrum of Nano Hydro 2 are detected at three positions: C_lattice_ at 530.3 eV [48] (5.8%), O–C(=O)–C [49]/N–C(=O)–C [43]/O–C(=O)–Ar [49] at 531.7 eV (13.6%), and O=C–O–C–O [49] at 532.8 eV (2.1%). Lattice oxygen atoms represent oxygen atoms within a bulk structure. In redox reactions, such as those used in this study for hydrogel and nanocomposite hydrogel synthesis, the lattice atoms can participate in electron transfer reactions [50]. Oxygen contamination of polyacrylamide gels during gel preparation has also been reported [51]. The higher atomic concentration of O_lattice_ in Nano Hydro 2 compared with Hydro 2 indicates lattice oxygen contributions from CNTs_OxCl_. Furthermore, the increased atomic concentration of the bonds detected at 531.7 eV in the spectrum of Nano Hydro 2 than those detected at 531.5 eV in the spectrum of Hydro 2 is due to the contributions of the O–C(=O)–Ar functionality. This confirms the incorporation of CNTs_OxCl_ in Nano Hydro 2 and reflects the aromatic nature of CNTs, as previously reported [52]. Furthermore, the detection of the anhydride functionality at 532.8 eV in Nano Hydro 2 confirms that functionalized CNTs are embedded in the developed nanocomposite hydrogels, as shown in Scheme 1.

**Figure 7:**
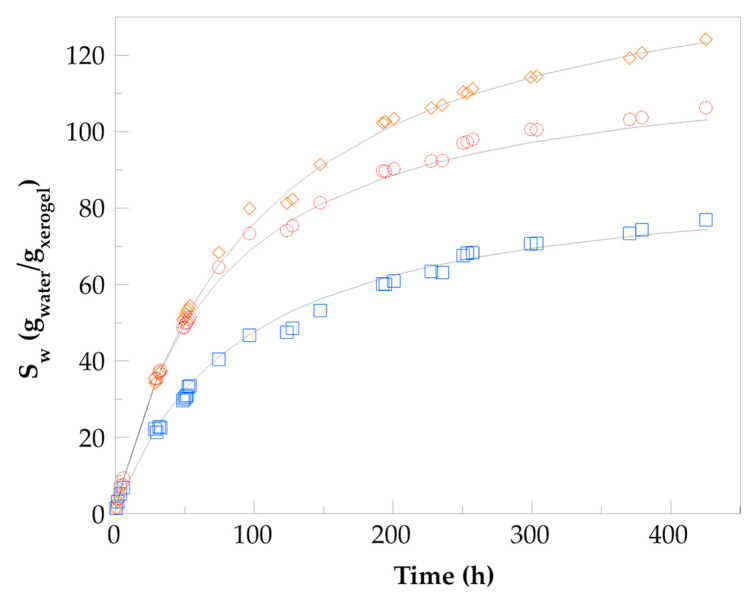
Swelling isotherms of the hydrogels Hydro 1 (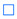), Hydro 2 (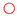), and Hydro 3 (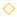).

**Figure 8:**
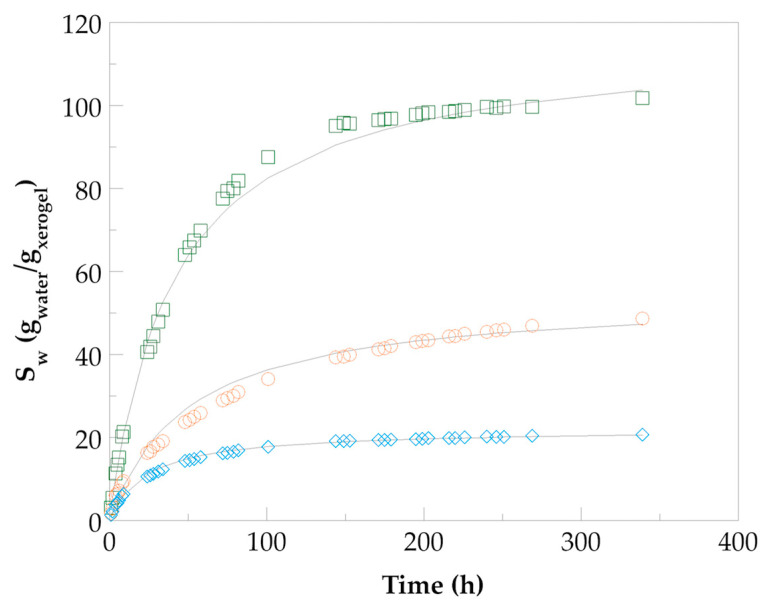
Swelling isotherms of the nanocomposite hydrogels Nano Hydro 1 (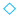), Nano Hydro 2 (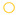), and Nano Hydro 3 (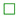).

Figure 7 and Figure 8 show the swelling isotherms of the AM–AA and AM–AA/CNTs_OxCl_ nanocomposite hydrogels, respectively, both exhibiting typical swelling profiles as a function of time [53]. At early times, the isotherms display a quasi-linear shape that gradually stretches asymptotically until the equilibrium–swelling limit is reached. The solid line represents the prediction of the swelling kinetic data, which are obtained by integrating the second-order kinetic model (Equation (8)) under the initial condition *W* = 0 at *t* = 0 to obtain the following:

(1) $$W=\frac{k\;W_{\infty }^{2}\;t}{1+k\;W_{\infty }\;t}\;$$

This equation can be simplified into an equation that predicts swelling as a function of time *t*:

(2) $$S_{w}=\frac{t}{t\left(m-1\right)+b}$$

where *m* and *b* are the slope and intercept, respectively, of a linear equation describing the evolution of $t\left(S_{w}+1\right)/S_{w}$ (calculated from experimental data) as a function of *t*, which can be obtained using a previously reported method [25]. The values of *m* and *b* can be obtained graphically and used to calculate the values of equilibrium–swelling $\left(S_{w\infty }\right)$ and *k* using the equations:

(3) $$m=\frac{S_{w\infty }+1}{S_{w\infty }}$$

(4) $$b=\frac{{\left(S_{w\infty }+1\right)}^{2}}{k\;S_{w\infty }^{2}}$$

Table 4 shows the measured $S_{w\infty }$ values alongside those predicted by the described model. The accuracy of the proposed model is excellent, with coefficients of determination (R^2^) of at least 0.9999 for both AM–AA hydrogels and AM–AA/CNTs_OxCl_ nanocomposite hydrogels. It is evident that the measured and predicted $S_{w}$ values are considerably close. Furthermore, *k* and $S_{w}$ increase with AA content for both the AM–AA hydrogels and AM–AA/CNTs_OxCl_ nanocomposite hydrogels. This behavior results from the ionization of the AA units. AA is a weak acid with a pKa of 4.66. Therefore, at pH > 4.66 the AA units ionize and the number of ions increases with increasing pH, promoting greater swelling [54]. However, the $S_{w}\;$ values of the AM–AA/CNTs_OxCl_ nanocomposite hydrogels are lower than those of the AM–AA hydrogels, suggesting that CNTs_OxCl_ induces additional crosslinking via NMBA. As detected in the XPS analysis, polymeric chains formed by the AA and AM units are grafted onto CNTs_OxCl_, constraining their mobility.

**Figure 9:**
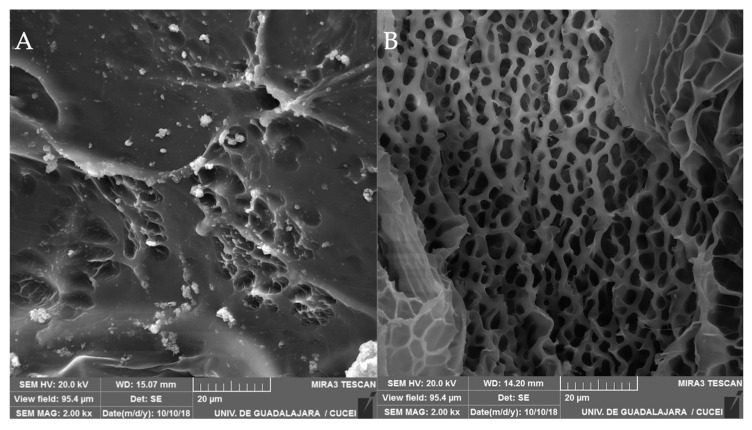
FE–SEM micrographs of the Nano Hydro 1 (**A**) and Hydro 1 (**B**).

Figure 9 shows the FE–SEM micrographs of Hydro 1 and Nano Hydro 1, both recorded with the same scale bars (20 μm). A distinctive morphological characteristic in both micrographs is the presence of pores. ImageJ software (version 1.53) was used to calculate the size of the detected pores, calibrated with the scale bar [55]. In the micrograph of Hydro 1, 150 pores are detected with an average size of 3.3 μm, whereas in the micrograph of Nano Hydro 1, 70 pores are observed with an average size of 5.5 μm. This increase in pore size in Nano Hydro 1 can be attributed to the higher concentration of carboxylate ions, which repel each other within the polymer network. As noted in the Raman spectra of the nanocomposite hydrogels (Figure 4), no evidence of dimer formation between the AA units is observed. This absence indicates that the number of carboxylate ions in the AM–AA/CNTs_OxCl_ nanocomposite hydrogels is higher than that in the AM–AA hydrogels.

Table 5 lists the Young moduli and the coefficient of determination R^2^ of the AM–AA hydrogels and AM–AA/CNTs_OxCl_ nanocomposite hydrogels measured at equilibrium swelling. The Young modulii of all nanocomposite hydrogels are higher than those of the corresponding hydrogels prepared with the same AA/AM composition. This strongly indicates that the polymeric chains formed by AA and AM units grafted onto CNTs_OxCl_ efficiently transfer the applied load to these nanostructures, thereby providing mechanical resistance to the nanocomposite hydrogel network, enhancing its mechanical performance.

**Figure 10:**
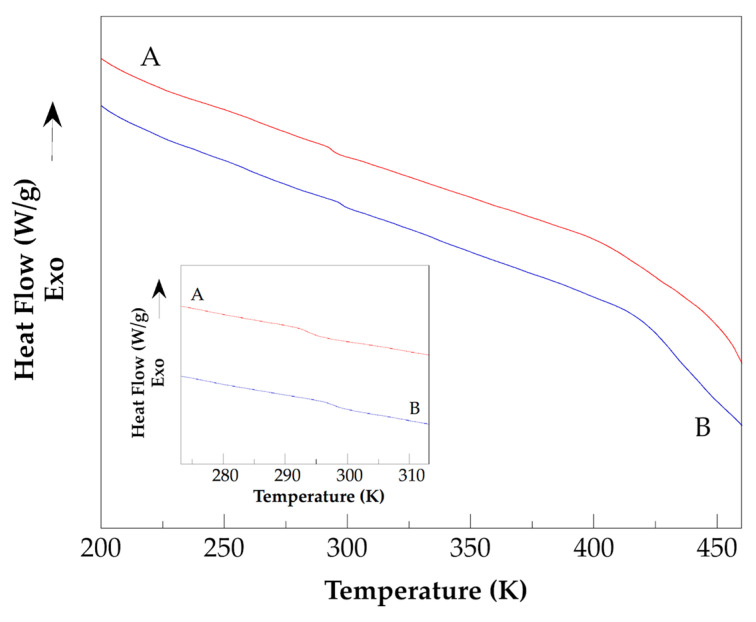
DSC thermograms of Hydro 1 (A), and Nano Hydro 1 (B).

Figure 10 shows the DSC thermograms of Hydro 1 (Figure 10A) and Nano Hydro 1 (Figure 10B). These thermograms depicts a range of temperature from 203.15 K to 463.15 K. The only thermal transition detected was a subtle transition due to the glass relaxation. As inset, DSC partial thermograms recorded from 273.15 K to 313.15 K was shown, in which a better appreciation of mentioned transition is observed. Table 5 presents the glass transition temperatures of all hydrogels studied. As the content of AA used to prepare both AM–AA hydrogels and AM–AA/CNTs_OxCl_ nanocomposite hydrogels increases, the $T_{g}$ was detected at higher temperatures. In addition, the $T_{g}$ of all AM–AA/CNTs_OxCl_ nanocomposite hydrogels was higher than that of the AM–AA hydrogels, both prepared at the same monomeric relation (wt%). The last result confirms the ability of the CNTs_OxCl_ to induce an additional crosslinking, because higher energy was needed to induce glass relaxation of the AM–AA/CNTs_OxCl_ nanocomposite hydrogels.

**Figure 11:**
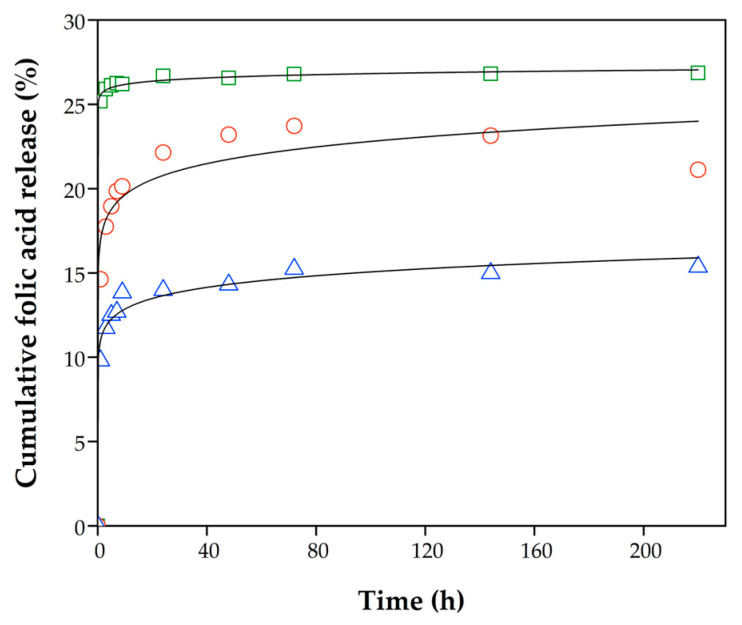
Folic acid release for Hydro 1 (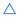), Hydro 2 (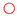), and Hydro 3 (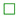).

**Figure 12:**
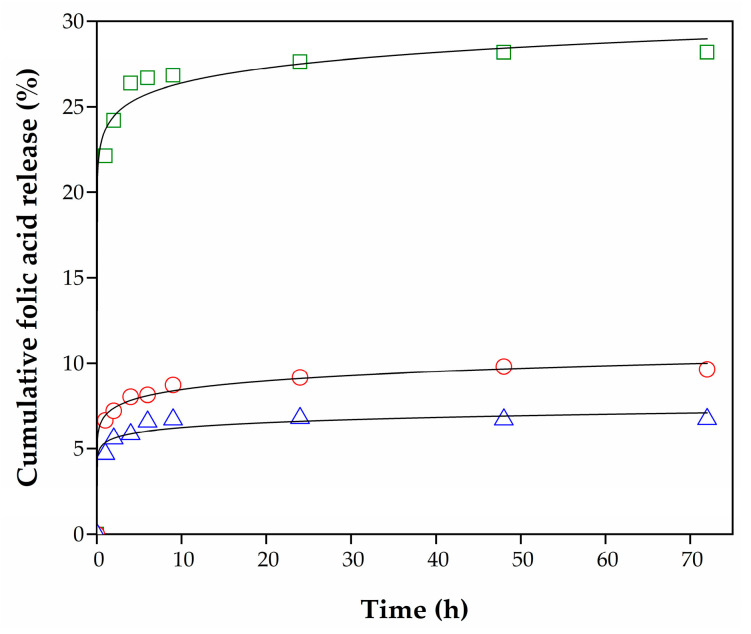
Folic acid release for Nano Hydro 1 (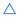), Nano Hydro 2 (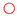), and Nano Hydro 3 (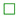).

Figure 11 and Figure 12 show the FA release profiles from the AM–AA hydrogels and AM–AA/CNTs_OxCl_ nanocomposite hydrogels, respectively. Both systems exhibit similar biphasic cumulative release behaviors, in which an initial rapid “burst release” phase is observed, followed by a plateau. As the AA content increases, the percentage of FA released also increases; however, the release from Hydro 1 and Hydro 2 is higher than that from Nano Hydro 1 and Nano Hydro 2, respectively. A similar trend is observed for Hydro 3 and Nano Hydro 3. Two factors influence these results: (i) the AA content in the polymeric network, and (ii) the number of pores in the AM–AA hydrogels and AM–AA/CNTs_OxCl_ nanocomposite hydrogels. A higher number of pores facilitate this release. To analyze the FA release data, nine kinetic models are employed: zero order, first order, Higuchi, Korsmeyer–Peppas, Hixson–Crowell, quadratic, Gompertz, Probit, and Weibull models. The goodness of fit of each model is evaluated using the following statistical criteria: (i) coefficient of determination (R^2^), (ii) mean square error (MSE), and (iii) model selection criterion (MSC) provided by DDSolver software (version 2.9.0) [56]. The best fit is considered for the model exhibiting an R^2^ value close to unity, a minimal MSE value, and maximal MSC value. Based on these criteria, the Weibull model (modified to suit a dissolution/release process) provides the best fit for the release data of FA from the AM–AA hydrogels and AM–AA/CNTs_OxCl_ nanocomposite hydrogels. The solid lines drawn over the experimental points shown in Figure 11 and Figure 12 correspond to the fit of the model. The Weibull model adapted to the release process is expressed using the following equation [57]:

(5) $$F=100\left\{1-e^{\left[\frac{-{\left(t-T_{i}\right)}^{b}}{a}\right]}\right\}$$

where F is the percentage of drug released, which is calculated from $M_{t}/M_{\infty }$. Here $M_{t}$ is the amount of drug released at time t, $M_{\infty }$ is the amount of drug loaded, a determines the time scale of the process, $T_{i}$ defines the lag time before the onset of the dissolution or release process (often being zero [58]), and b is the shape parameter. This parameter characterizes the curve as either exponential (b = 1) (Case 1); sigmoid, S-shaped (b > 1) (Case 2); or with a steeper initial slope followed by exponential behavior (b < 1) (Case 3). Table 6 lists the values of the Weibull parameters for the AM–AA hydrogels and AM–AA/CNTs_OxCl_ nanocomposite hydrogels and the R^2^ values. The calculated R^2^ values range from 0.973 (for Hydro 2) to 0.999 (for Hydro 3), indicating that a reasonable adjustment is achieved with the Weibull model. For all samples, b < 1, indicating Case 3 behavior under the prediction of the Weibull model. Furthermore, considering the suggestion of Langenbucher [57] to replace parameter a with $a={\left(T_{d}\right)}^{b}$, where $T_{d}$ is the dissolution/release time, and assuming that $T_{i}$ = 0, Equation (5) can be modified as follows:

(6) $$F=100\left[1-e^{-{\left(\frac{t}{T_{d}}\right)}^{b}}\right]$$

where $T_{d}$ and b are constants that depend on the specific release mechanisms. When b≤0.75, the release mechanism is attributed to Fick diffusion in either Ecluidian or fractal spaces [59]. Based on the fact that all values of b<0.75 and the calculated values of $T_{i}$ (Weibull model) are negligible (Equation (5)), it is possible to consider that FA release from the AM–AA hydrogels and AM–AA/CNTs_OxCl_ nanocomposite hydrogels follows Fickian diffusion. It is important to mention that our study has limitations associated with the difficulty of controlling the dispersion of the CNTs_OxCl_ in the network of synthesized nanocomposite hydrogels.

The strategy used in this study to evaluate the role of FA in the potential use of nanocomposite hydrogels prepared with CNTs is different from that reported in other studies, which evaluate the release of anticancer drugs from a hybrid material reinforced with CNTs conjugated with FA [60,61]. Therefore, this opens the possibility of developing new studies that explore the simultaneous release of FA and an anticancer drug from a polymeric nanocomposite.

## 3. Materials and Methods

### 3.1. Materials

Acrylamide (AM) (99.9% purity) was purchased from IBI Corp. (Armonk, NY, USA). N,N’–methylene–bis–acrylamide (NMBA) (99%), acrylic acid (AA) (99%), potassium persulfate (KPS) (99%), oxalyl chloride (98%), dichloromethane (ACS reagent), hydrochloric acid (37.5%), triethylamine (Et_3_N) (99%), and FA (97%) were provided by Sigma–Aldrich (Saint Louis, MO, USA). Argon gas (99.998%) was obtained from PRAXAIR (Guadalajara, Mexico). Nitrogen gas (99.99%) was obtained from INFRA (Guadalajara, Mexico). Alumina boats were purchased from Alfa Aesar (Tewksbury, MA, USA). Anhydrous ethanol (99.8%) was obtained from Fermont (Monterrey, Mexico). Ferric nitrate nonahydrate (Fe(NO_3_)_3_·9H_2_O) (98.2%), sodium bisulfite (NaHSO_3_) (ACS reagent), and nitric acid (70%) were acquired from Golden Bell (Guadalajara, México). Double–distilled water was obtained from Productos Selectropura (Guadalajara, Mexico). All the mentioned chemical reagents were employed without further purification.

### 3.2. Synthesis, Purification, Partial Oxidization, and Chemical Functionalization of CNTs

CNTs were synthesized via CVD following our previously reported method [62]. Pristine CNTs were purified using overheated steam following our previously reported methodology [24]; the purified CNTs are denoted as CNTs_pur_. CNTs_pur_ were washed repeatedly with water and dried in an oven at 333.15 K for 24 h, followed by acidic treatment under reflux to partially oxidized them. Acidic treatment involved placing 1 g of CNTs_pur_ in a 250 cm^3^ glass Soxhlet apparatus together with 100 cm^3^ of a 4 M nitric acid solution. The mixture was heated until seven reflux cycles occurred, followed by washing with double–distilled water until obtaining a pH of 7. The product (partially oxidized CNTs) was dried in an oven at 333.15 K to a constant weight and was named CNTs_poxi_. The content of the hydroxyl and acidic carboxyl groups inserted into the walls of CNTs_poxi_ was calculated via previously reported indirect back-titration methods, where a hydrochloric acid solution was reacted with excess sodium bicarbonate to determine the amount of acidic carboxyl groups, or with an excess sodium hydroxide solution to calculate the amount of hydroxyl groups [28,29]. CNTs_poxi_ were treated with OxCl to create acyl chloride functionalities on their walls, following our previously reported chemical procedure [35]. Briefly, 0.5 g of CNTs_poxi_ was placed in a 100 mL glass reactor partially immersed in an oil bath at 323.15 K, followed by the addition of 95 μL of Et_3_N dissolved in 5 mL of dichloromethane. The dispersion was stirred under a nitrogen atmosphere. After 15 min, 325 μL of OxCl dissolved in 25 mL of dichloromethane was added dropwise with a syringe and allowed to react for at least 1 h. Subsequently, the solvent was completely evaporated at 333.15 K, and the product was named CNTs_OxCl_ and stored in a desiccator to remove any moisture.

### 3.3. Synthesis and Purification of the AM–AA Hydrogels and AM–AA/CNTs_OxCl_ Nanocomposite Hydrogels

AM–AA and AM–AA/CNTs_OxCl_ nanocomposite hydrogels were synthesized via redox polymerization. For AM–AA hydrogel synthesis, a mixture of potassium persulfate (0.05 wt%) and sodium bisulfite (0.05 wt%) was used as the redox initiator, while N,N’–methylene–bis(acrylamide) (0.1 wt%) was employed as the cross-linker agent. Furthermore, a mixture prepared from three different weight ratios of AM and AA (7/3, 8/2, or 9/1 *w*/*w* of AM/AA) was used as the co-monomeric mixture (10 wt%) with 89.8 wt% water. For the synthesis of the AM–AA/CNTs_OxCl_ nanocomposite hydrogels, similar formulations were employed, with 0.1 wt% CNTs_OxCl_ and a reduced water content (89.7 wt%). Table 7 lists the identification of samples of prepared hydrogels as a function of its formulation. The preparation of the hydrogels with or without CNTs_OxCl_ was conducted in a similar way; for preparing the AM–AA hydrogels, aqueous solutions of the monomer mixture of AM/AA, NMBA, and the initiator system KPS/NaHSO_3_ were prepared separately. Each solution was bubbled with N_2_ for 5 min, after which, the three solutions were mixed and placed in a glass tube. The resulting mixture was stirred for 5 min and partially submerged in a water bath at 313.15 K; the hydrogel synthesis was performed for 24 h.

To synthesize the AM–AA/CNTs_OxCl_ nanocomposite hydrogels, aqueous solutions of AM/AA, NMBA, and KPS/NaHSO_3_ were prepared separately. The three solutions were bubbled with N_2_ gas for 5 min, after which, an amount of CNTs_OxCl_ was added to the monomeric solution and the dispersion was stirred in an ultrasonic bath (3510R–MTH Bransonic Ultrasonic Cleaner, Danbury, CT, USA) operated at 42 kHz ± 6%, and 100 W for 15 min to ensure homogenous dispersion of the functionalized CNTs. The dispersion, together with the NMBA and KPS/NaHSO_3_ solutions were mixed and stirred in a glass tube for 10 min, then immersed in a water bath at 313.15 K to initiate nanocomposite hydrogel synthesis. The reaction was allowed to proceed for 24 h.

After polymerization, the hydrogels were recovered by breaking each glass tube. Then, each hydrogel was cut into disks (1.3 cm i.d., 1.0 cm length) and washed to remove any unreacted residual monomers and other chemical agents, followed by immersion in a 500 mL beaker containing 250 mL of water (water was renewed every 2 d for at least 8 d) until the pH reached 7. The disks were then dried at room temperature for at least 7 d to a constant weight. After this, they were placed in an oven at 308.15 K until the start of their characterization to prevent them from becoming damp from the ambient humidity. The dry gel disks (xerogels) were characterized using various experimental techniques and employed in swelling studies.

### 3.4. Characterization of CNTs, AM–AA Hydrogels, and AM–AA/CNTs_OxCl_ Nanocomposite Hydrogels

#### 3.4.1. Transmission Electron Microscopy (TEM)

Purified, partially oxidized, and functionalized CNTs were examined using a transmission electron microscope (JEOL, Peabody, MA, USA; model 1010) operated at 200 kV. The samples were completely dried in an oven at 343.15 K for at least 72 h prior to analysis. Subsequently, a small amount of the sample (approximately 0.010 g) was dispersed in 2 mL of acetone, followed by sonication for 5 min at room temperature. An aliquot was drop-cast onto a copper grid using a Pasteur pipette and dried under a 60 W solar lamp for 20 min prior to examination.

#### 3.4.2. Raman Spectroscopy

The partially oxidized CNTs, functionalized CNTs, AM–AA hydrogels, and AM–AA/CNTs_OxCl_ nanocomposite hydrogels were analyzed via Raman spectroscopy using a Dilor (Villeneuve–d’Ascq, France) Lab Raman II spectrometer equipped with a HeNe laser. The spectrometer was operated at 20 mW with an excitation wavelength of 632.8 nm. A 50× objective with a spot area of 2 mm and 2 cm^−1^ error was used to perform the measurements.

#### 3.4.3. X-Ray Photoelectron Spectroscopy (XPS)

The AM–AA and AM–AA/CNTs_OxCl_ nanocomposite hydrogels were analyzed via XPS using a Phoibos 150 spectrometer with a one-dimensional detector 1D–DLD (SPECS, Berlin, Germany) and an XR50 M monochromatic Al Kα_1_ (*hv* = 1486. eV) X-ray source. Prior to analysis, the samples were deposited on a steel sample holder using copper tape to avoid the interface from the carbon tape. Subsequently, they were dried in a vacuum oven for 48 h and then placed in the prechamber (base pressure, 4.2 × 10^−10^ mbar). Measurements were acquired at 150 W, with an electron take-off angle of 90°, pass energy of 10 eV, and step size of 0.1 eV. A flood gun was employed for charge neutralization, and spectra were calibrated to the C–C binding energy.

#### 3.4.4. Field-Emission Scanning Electron Microscopy (FE–SEM)

The AM–AA and AM–AA/CNTs_OxCl_ nanocomposite hydrogels were examined using a MIRA 3LU field-emission scanning electron microscope (FE–SEM) (Tescan, Brno, Czech Republic). Prior to analysis, the samples were dried in an oven at 333.15 K for 72 h, after which, approximately 0.01 g of each sample was mixed with 1 cm^3^ of acetone at room temperature (~298.15 K), sonicated for 5 min, and drop-cast onto a copper grid using a Pasteur pipette. After acetone evaporation, the sample was introduced to an SCD004 golden evaporator (BalTec AG, Pfäffikon, Switzerland), and a thin gold layer was applied through a 20 s electrodeposition process before FE–SEM examination.

#### 3.4.5. Swelling Tests

The swelling kinetics of the AM–AA and AM–AA/CNTs_OxCl_ nanocomposite hydrogels were determined gravimetrically. The dry disks (xerogels) were weighed and immersed in double-distilled water in sealed 300 mL plastic containers and the swelling tests were performed at room temperature (~298.15 K). At specific time points, the disks were recovered, blotted with absorbent tissue paper to remove any excess water, and weighed. The swelling ratio (*S_w_*) was determined as follows:

(7) $$S_{w}=\frac{m-m_{0}}{m_{0}}\;\left(\frac{g_{water}}{g_{xerogel}}\right)$$

where $m_{0}$ and m are the masses of the xerogel and hydrogel, respectively, at time *t*. In this equation $g_{water}$ is gram of water, while $g_{xerogel}$ is gram of xerogel. The weights of the three disks were measured at the same time t to record the average swelling value and swelling was followed until equilibrium. Data were fitted to a second-order kinetic model as follows [63]:

(8) $$\frac{dW}{dt}=k{\left(W_{\infty }-W\right)}^{2}$$

where k is the rate constant, W is the water uptake at time t, and $W_{\infty }$ is the equilibrium water uptake. The following equation, together with Equations (1), (2) and (4), was used to determine *k*:

(9) $$W=\frac{m-m_{0}}{m}$$

where the values of $m_{0}$ and m are determined at time zero and time *t*, respectively.

#### 3.4.6. Mechanical Tests

Compression tests were performed on AM–AA hydrogel and AM–AA/CNTs_OxCl_ nanocomposite hydrogel disks swollen to equilibrium at room temperature (~298.15 K) using an ARES G2 rheometer (TA Instruments, New Castle, DE, USA) operated in parallel dish mode. The disks (2.7 cm i.d. and 1.2 cm thickness) were compressed and the Young’s modulus (*E*) was determined from the slope of the initial zone of the stress–strain curve (<5% strain) using the following equation:

(10) $$\tau =-E\left(\lambda -1\right)$$

where *λ* is the deformation ratio of the deformed (*h*) to the initial thickness (*h*_0_) of the hydrogel disks, and τ is the applied stress. The measurements were repeated six times for each type of hydrogel or nanocomposite hydrogel studied, and the numerical average is reported.

#### 3.4.7. UV–Vis Spectroscopy

The in vitro FA release from the AM–AA and AM–AA/CNTs_OxCl_ nanocomposite hydrogels was determined via UV–Vis spectroscopy. The release profile of FA was determined by considering the absorbance as a function of time using an S–2150 UNICO spectrometer (South Brunswick, NJ, USA), after establishing a calibration curve at λ = 295 nm. To load FA, xerogels (0.2 g) were immersed in 50 mL of an aqueous solution of 100 ppm FA for 72 h at room temperature (~298.15 K) under constant stirring to achieve FA caption during hydrogel swelling. Measurements were performed in triplicate in an aqueous medium, avoiding exposure to visible light.

#### 3.4.8. Differential Scanning Calorimetry (DSC)

The AM–AA and AM–AA/CNTs_OxCl_ nanocomposite hydrogels were analyzed via DSC using a Q100 calorimeter (TA Instruments, New Castle, DE, USA). DSC thermograms were obtained following a heating program from 183.15 K to 473.15 K at heating rate of 5 K/min using a nitrogen flow rate of 50 cm^3^/min to maintain an inert atmosphere in the sample cell of the DSC equipment. The masses of the samples were in the range of 3 to 5 mg. Two heating scans were obtained, and the second scan is reported. The glass–transition temperature ($T_{g}$) of all prepared hydrogels were determined using the first derivative criteria.

## 4. Conclusions

AM–AA hydrogels and AM–AA/CNTs_OxCl_ nanocomposite hydrogels were prepared and characterized, and their ability to release FA was evaluated. Helical and straight CNTs were obtained via the CVD method and used in the preparation of nanocomposite hydrogels. The hydroxyl and carboxyl group contents on CNTs_poxi_ were quantified. TEM and Raman spectroscopy confirmed the presence of MWCNTs in CNTs_poxi_ and CNTs_OxCl_, while Raman analysis also suggested the successful chemical functionalization of CNTs_OxCl_. Raman and XPS analyses verified the synthesis of AM–AA hydrogels and AM–AA/CNTs_OxCl_ nanocomposite hydrogels. Raman spectroscopy revealed the presence of dimers formed by the self-association of the carboxylic acid groups in the AM–AA hydrogels, whereas XPS revealed that polymer chains composed of AM and AA units were grafted to CNTs_OxCl_ in the AM–AA/CNTs_OxCl_ nanocomposite hydrogels. The swelling kinetics of the developed hydrogels were successfully adjusted using a second-order equation. Compared with the AM–AA/CNTs_OxCl_ nanocomposite hydrogels, the AM–AA hydrogels showed greater swelling, whereas the Young’s moduli and glass transition temperatures of the AM–AA/CNTs_OxCl_ nanocomposite hydrogels were higher than those of the AM–AA hydrogels. FE-SEM micrographs revealed surface pores in all synthesized hydrogels, with larger pores in the AM–AA/CNTs_OxCl_ nanocomposite hydrogels. This was attributed to the repulsion between the carboxylate ions within the polymer network of the nanocomposite hydrogels. The Weibull model adequately adjusted the FA release data recorded for all the studied hydrogels, whereas the release mechanism was driven by Fickian diffusion. Our study provides findings that can be used in the nanomedicine field for the design of nanocarriers suitable to transport anticancer drugs.

## Figures and Tables

**Table 1:** Wavenumbers of the D-, G-, and G′-bands, as well as areas of the G-band and scattering intensity ratios (I_D_/I_G_) of CNTs_poxi_ and CNTs_OxCl_.

Samples	D-Band (cm^−1^)	G-Band (cm^−1^)	G′-Band (cm^−1^)	G-Band Area (u. a.)	I_D_/I_G_
CNTs_poxi_	1233	1485	2436	19.49	0.81
CNTs_OxCl_	1235	1466	2461	41.43	1.56

**Table 2:** Identification of Raman bands of the AM-AA hydrogels.

Band	Assignment
a	CH_2_ rocking
b	C–C skeletal stretching
c	NH_2_ wagging
d	CH_2_ wagging
e	amide I, C=O stretching
f	C–COOH stretching
g	COOH dimer stretching
h	CH_2_ rocking
i	C-CH_2_ stretching
j	C–O stretching coupled with O–H in-plane bending
k	–CCO stretching

**Table 3:** Identification of Raman bands of the AM–AA/CNTs_OxCl_ nanocomposite hydrogels.

Band	Assignment
A	CH_2_ rocking
B	C–C skeletal stretching
C	CH_2_ wagging
D	amide I, C=O stretching
E	CH_2_ rocking
F	C-CH_2_ stretching
G	–CCO stretching
H	NH_2_ wagging/D-band

**Table 4:** Predicted equilibrium–swelling and swelling rate constants obtained from the second-order swelling kinetic model, and experimentally measured equilibrium–swelling for the AM-AA hydrogels and AM-AA/CNTs_OxCl_ nanocomposite hydrogels.

Samples	$\;\;\;\;S_{w\infty }^{§}\;\left(\frac{g_{water}}{g_{xerogel}}\right)$	$\;\;\;\;S_{w\infty }^{†}\;\left(\frac{g_{water}}{g_{xerogel}}\right)$	$\;\;\;k\;\left(\frac{g_{hydrogel}}{g_{water}h}\right)$
Hydro 1	77.0	74.4	1.0
Hydro 2	106.3	103.1	1.5
Hydro 3	124.2	123.4	1.7
Nano Hydro 1	20.6	20.5	1.0
Nano Hydro 2	48.6	47.3	1.1
Nano Hydro 3	101.3	103.6	2.9

^§^ Values obtained from experimental measurements. ^†^ Values recorded from the second-order swelling kinetic model fitting.

**Table 5:** Young moduli, coefficient of determination (R^2^) and glass transition temperatures of the AM–AA hydrogels and AM–AA/CNTs_OxCl_ nanocomposite hydrogels recorded at equilibrium swelling.

Samples	*E* (kPa)	Coefficient of Determination (R^2^)	$T_{g}$ (K)
Hydro 1	6.6	0.994	293.35
Hydro 2	7.6	0.994	296.05
Hydro 3	8.8	0.994	305.35
Nano Hydro 1	9.2	0.996	297.55
Nano Hydro 2	10.3	0.990	298.35
Nano Hydro 3	10.6	0.983	306.75

**Table 6:** Weibull model parameters and coefficient of determination (R^2^) for the AM-AA hydrogels and AM-AA/CNTs_OxCl_ nanocomposite hydrogels.

Samples	Parameters of the Weibull Model			Coefficient of Determination (R^2^)
	$T_{i}$	a	b	
Hydro 1	0.96	8.0	0.057	0.993
Hydro 2	0.98	4.9	0.054	0.973
Hydro 3	0.96	3.3	0.009	0.999
Nano Hydro 1	0.99	16.6	0.042	0.987
Nano Hydro 2	0.75	13.1	0.074	0.997
Nano Hydro 3	0.97	3.4	0.037	0.998

**Table 7:** Identification of samples of the AM-AA hydrogels and AM-AA/CNTs_OxCl_ nanocomposite hydrogels according to their formulation in wt%.

Samples	AM	AA	NMBA	Na_2_SO_3_	KPS	CNTs_OxCl_	H_2_O
Hydro 1	9	1	0.1	0.05	0.05	0	89.8
Hydro 2	8	2	0.1	0.05	0.05	0	89.8
Hydro 3	7	3	0.1	0.05	0.05	0	89.8
Nano Hydro 1	9	1	0.1	0.05	0.05	0.1	89.7
Nano Hydro 2	8	2	0.1	0.05	0.05	0.1	89.7
Nano Hydro 3	7	3	0.1	0.05	0.05	0.1	89.7

## Data Availability

The data presented in this study are available upon request from the corresponding author.

## Abbreviations

The following abbreviations are used in this manuscript:

AM	Acrylamide
AA	Acrylic acid
CNTs	Carbon nanotubes
CVD	Chemical vapor deposition
TEM	Transmission electron microscopy
XPS	X-ray photoelectron spectroscopy
FE-SEM	Field-emission scanning electron microscopy
SWCNTs	Single–walled carbon nanotubes
MWCNTs	Multi–walled carbon nanotubes
DDS	Drug delivery systems
UCST	Upper critical solution temperature
PPy	Polypyrrole
5-FU	5–fluorouracil
NIR	Near–infrared
PAAm	Polyacrylamide
PAAc	Polyacrylic acid
IPN	Interpenetrating network
